# PREPARING “EXPERIENCED” CAREGIVERS FOR FUTURE CARE: PERSPECTIVES FROM FORMER CAREGIVERS OF PARENTS WITH DEMENTIA

**DOI:** 10.1093/geroni/igad104.1370

**Published:** 2023-12-21

**Authors:** Emily Mroz, Amanda Piechota, Talha Ali, Tara Matta-Singh, Anissa Abboud, Shubam Sharma, Terri Fried, Joan Monin

**Affiliations:** Yale School of Medicine, New Haven, Connecticut, United States; Yale School of Public Health, New Haven, Connecticut, United States; Tufts University, Medford, Massachusetts, United States; University of South Florida, Tampa, Florida, United States; Yale School of Public Health, New Haven, Connecticut, United States; Kennesaw State University, Kennesaw, Georgia, United States; Yale School of Medicine, New Haven, Connecticut, United States; Yale School of Public Health, New Haven, Connecticut, United States

## Abstract

Providing care for more than one care partner across adulthood is becoming increasingly common, resulting in a growing number of ‘experienced’ caregivers (those who have been caregivers before and are anticipating or engaging in caregiving roles again). Caregivers have opportunities to develop caregiving skills and experience positive and negative psychological consequences that persist after care roles have ended. Little is known about how these factors shape preparedness in the large and growing group of former adult-child caregivers of parents living with dementia (PLWD) who anticipate future caregiving roles. We conducted a qualitative study of 32 interviews with midlife former caregivers of now-deceased PLWD. Consensual Qualitative Research and grounded theory techniques guided study design and analysis. Analyses demonstrated that preparedness for future caregiving roles was shaped by personal narratives from former caregiving roles across two dimensions: insights and confidence. All caregivers described insights (i.e., caregiving self-conduct, care systems and resources, and relating with a care partner) that contributed to their preparedness. Though some former caregivers also described a positive (i.e., boosted or sustained) sense of confidence, others described a diminished (i.e., restricted or impeded) sense of confidence. Findings suggest that for some former caregivers, past experiences offer cumulative advantages; for others, past experiences may contribute to apprehension towards, or rejection of, future caregiving roles. Entering new roles with diminished confidence may have negative consequences for caregivers’ and care partners’ wellbeing. This presentation will conclude with suggestions for resources that address both insight-based and confidence-based preparedness and are tailored to experienced caregivers.

